# Growth Differentiation Factor 5 Improves Neurogenesis and Functional Recovery in Adult Mouse Hippocampus Following Traumatic Brain Injury

**DOI:** 10.3389/fneur.2018.00592

**Published:** 2018-07-23

**Authors:** Hongjie Wu, Jing Li, Dongxiao Xu, Qiansheng Zhang, Tao Cui

**Affiliations:** Department of Neurosurgery The First Affiliated Hospital and College of Clinical Medicine of Henan University of Science and Technology, Luoyang, China

**Keywords:** growth differentiation factor 5, hippocampus, neurogenesis, traumatic brain injury, functional recovery

## Abstract

The aim of this study was to investigate the therapeutic effect of growth differentiation factor 5 (GDF-5) on traumatic brain injury (TBI) in mice. We utilized a controlled cortical impact to establish a mouse TBI model, and then stereotaxically administered 25 or 100 ng GDF-5 into the bilateral hippocampal dentate gyrus (DG) of each of the animals. Seven days after the injury, some of the animals were sacrificed for immunohistochemical and immunofluorescence examination of 5-bromo-2′-deoxyuridine (BrdU), Sox-2, doublecortin (DCX) and phosphorylated cAMP response element binding protein (p-CREB). Dendrite quantification was also performed using DCX positive cells. Activation of newborn neurons was assessed 35 days after the injury. The remaining animals were subjected to open field, Y maze and contextual fear conditioning tests 2 months after TBI. As a result, we found that post-injury stereotaxical administration of GDF-5 can improve neural stem cell proliferation and differentiation in the DG of the hippocampus, evidenced by the increase in BrdU, Sox-2, and DCX-labeled cells, as well as the improvement in dendrite arborization and newborn neuron activation in response to GDF-5 treatment. Mechanistically, these effects of GDF-5 may be mediated by the CREB pathway, manifested by the recovery of TBI-induced dephosphorylation of CREB upon GDF-5 administration. Behavioral tests further verified the effects of GDF-5 on improving cognitive and behavioral dysfunction after TBI. Collectively, these results reveal that direct injection of GDF-5 into the hippocampus can stimulate neurogenesis and improve functional recovery in a mouse TBI model, indicating the potential therapeutic effects of GDF-5 on TBI.

## Introduction

Traumatic brain injury (TBI) is the leading cause of death and disability around the world, which has been regarded as a major public health care burden borne by millions of people annually (1). TBI is a heterogeneous disease; its pathophysiology includes primary and secondary brain injury, which occurs at the time of trauma and in the hours and days following the primary injury (2). Secondary injury results from a cascade of molecular injury mechanisms, which consequently damages neurons that were unharmed in the primary injury and leads to other complications that can further exacerbate the brain injury (3). Therefore, it is widely accepted that secondary brain injury plays a major role in brain damage and brain death resulting from TBI, and thus needs to be taken seriously (4).

In clinics, however, a current major challenge associated with secondary brain injury of TBI is a lack of effective treatments due to the complex and heterogeneous nature of the injury (5). A number of therapeutic strategies have been the focus of extensive preclinical work into the development of neuroprotective and/or neurogenic treatments for secondary brain injury in TBI, but none of them have demonstrated clear benefits in clinical trials (6). Recently, with the documented findings of neurogenesis in adult humans, a novel potential therapeutic strategy has emerged with the goal of enhancing post-injury neurogenesis and consequently improving functional recovery after TBI (7). To achieve this goal, multiple therapeutic modalities, including small molecules, biologics, and stem cell-based therapies, have been exploited in *in vivo* experiments (8). Based on published data, there appears to be good prospects for targeting crucial signaling pathways that coordinate and regulate neurogenesis (9).

It is well-known that the dentate gyrus (DG) of the hippocampus is host to neurogenesis, where neural stem cells (NSCs) give rise to fully functional neurons (10). In recent years, a number of signaling pathways have been identified being involved in the neurogenic process, of which signaling among the members of the transforming growth factor beta (TGF-β) superfamily is a principal regulator (11). Although there is no absolute certainty, accumulating evidence has demonstrated the positive role of the members of the TGF-β superfamily in neuronal survival and the differentiation of hippocampus-derived NSCs (12, 13). Therefore, TGF-β superfamily signaling may present a promising target for interventions to improve neurogenesis, thereby promoting brain repair after TBI.

Growth differentiation factor 5 (GDF-5) is a member of the TGF-β superfamily (14). Previous studies have demonstrated that it exerts a direct neurotrophic effect on dopaminergic neurons, as well as a protective effect against kainic acid (KA)-induced injury on hippocampal neurons (14, 15). Hence, we hypothesized that increasing the level of GDF-5 in the brain could stimulate neurogenesis in the hippocampus and consequently improve functional recovery. To test this hypothesis, in this study we directly administered GDF-5 into the brains of mice subjected to TBI and investigated its effects on NSC proliferation, immature neuronal survival and differentiation, and recovery of hippocampal functions following the injury.

## Methods

### Animals and reagents

Adult male C57BL/6 mice (weighing 22–28 g, aged 8–12 weeks) were obtained from Shanghai Laboratory Animal Center, Chinese Academy of Sciences (Shanghai, China). All animal handling procedures were performed in accordance with the Chinese guidelines for animal welfare. The animal study protocol was approved by the Animal Ethics Committee of Henan University of Science and Technology.

Recombinant mouse GDF-5 was obtained from R&D Systems (Minneapolis, MN, USA). 5-bromo-2′-deoxyuridine (BrdU) was from Thermo Fisher Scientific (Waltham, MA, USA). The antibody against BrdU was purchased from Abcam (Cambridge, UK; AB6326). The antibodies against doublecortin (DCX) and Sox-2 were from Santa Cruz Biotechnology (Dallas, TX, USA; sc-8066 and sc-17320). The antibodies against phosphorylated cAMP response element binding protein (p-CREB, Ser 133), NeuN, and c-Fos were from Millipore (Billerica, MA, USA; 06-519, MAB377, and PC05).

### Establishment of mouse TBI model and GDF-5 treatment

The diagram of study design is shown in Supplementary Figure 1. A total of 84 C57BL/6 mice were randomly divided into four experimental groups with 21 mice per group: a sham control group (Sham); a model group (TBI); a model group treated with low-dose GDF-5 (25 ng/animal) (TBI + 25 ng GDF-5); a model group treated with high-dose GDF-5 (100 ng/animal) (TBI + 100 ng GDF-5). Prior to experiments, all animals were housed in a temperature- and humidity-controlled (22–25°C; 45–60%) room with a 12-h light/dark cycle. Food and water were available *ad libitum*.

Moderate controlled cortical impact (CCI) injury was performed according to the method previously reported (16). The details are described in the Supplementary Information. Right after induction of TBI, by using a Hamilton syringe, GDF-5 (25 or 100 ng/μl saline solution, 1 μl in volume) was stereotaxically administered into the hippocampal DG area of the impacted hemisphere in animals of the two GDF-5-treated groups [stereotaxic coordinates: anterior-posterior (AP) = −2.2 mm, medial-lateral (ML) = 1.6 mm, and dorsal-ventral (DV) = 2.0 mm at an infusion rate of at 0.25 μl/min]. The doses were chosen according to previous literature (15). Animals in the Sham and TBI groups received normal saline of equal volume. After treatment, the skin incision was sutured closed. During the whole surgical procedure, a heating pad was used to maintain the animals at 36–37°C.

### BrdU labeling of proliferating cells

One hour after TBI surgery, 13 mice from each experimental group received one intraperitoneal injection of BrdU (50 mg/kg) and the injection was continued for 6 days. On day 7, eight of these mice were deeply anesthetized with a mix of ketamine and xylazine (ketamine: 100 mg/kg, xylazine: 10 mg/kg, intraperitoneal injection) and sacrificed, and then transcardially flush-perfused with cold phosphate buffer (PBS). The brains were removed and processed for immunohistochemical (IHC) and immunofluorescence (IF) staining and analyses. The remaining five animals in the group were sacrificed after 4 weeks (on day 35) and their hippocampus samples were subjected to IF staining to investigate the activation of newborn neurons.

### Immunohistochemical staining and analysis

The brains of mice were removed and fixed in phosphate-buffered 4% paraformaldehyde (pH 7.4). Then, the fixed samples were sectioned in the coronal plane at 40 μm thickness using a cryomicrotome (Leica, Wetzlar, Germany). The obtained sections were incubated with 0.01 M sodium citrate buffer (pH 6.0) for 30 min at 90°C and then washed with Tris-buffered saline with Tween (TBST). Next, the sections were blocked in 10% goat serum for 1 h and then incubated with anti-BrdU (1:1,000), anti-DCX (1: 500), anti-Sox-2 (1:200), or anti-p-CREB (1:1,000) overnight at 4°C. Staining was revealed using biotinylated secondary antibodies and avidin-biotin complex (ABC) peroxidase standard staining kit (Thermo Fisher Scientific) with diaminobenzidine (DAB, Thermo Fisher Scientific). Immunopositive cells were quantified using Fiji ImageJ software (https://fiji.sc/). The total number of BrdU, DCX, and Sox-2-immunopositive cells (BrdU^+^, DCX^+^, and Sox-2^+^) per DG was estimated based on the method described previously (17), while p-CREB immunoreactivity in the granule cell layer (GCL) of the DG was quantified by using the IHC Profiler plugin for ImageJ according to the procedures reported by Varghese et al. (18). For each marker, 5 sections of the DG from an individual animal were randomly selected and analyzed by the same observer, who was unware of animal assignments.

### Immunofluorescence staining and analysis

On day 7, the obtained hippocampal sections were subjected to IF staining using combinations of primary antibodies against BrdU and Sox-2, followed by immunodetection using species-matched secondary antibodies coupled to Alexa Fluor 568 and 488 (#A-11077 and #A-11055, Thermo Fisher Scientific, 1:1,000). In addition, 4, 6-diamidino-2-phenylindole (DAPI, 5 μg/ml, Sigma-Aldrich, St. Louis, MO) was used to counterstain nuclei. On day 35, the obtained sections were stained for NeuN, BrdU, and c-Fos using the corresponding primary antibodies, and detected with respective secondary antibodies conjugated with Alexa Fluor 488, 568, and 350 (#A-11001, 1:2,000; #A-11077, 1:1,000; #A-11046, 1:1,000; Thermo Fisher Scientific). For quantitative analysis, z-stack fluorescence images were acquired with a confocal laser scanning microscope (LSM 710, ZEISS Microscopy, Jena, Germany) using a multi-track configuration. Immunostained cells in every sixth section throughout the DG were counted (BrdU^+^/Sox-2^+^ cells for day 7 and NeuN/BrdU/c-Fos positive (NeuN^+^/BrdU^+^/c-Fos^+^) cells for day 35). The density of immunostained cells was calculated by dividing the total cell number by the corresponding volume of the DG.

### Dendrite quantification

Dendrite quantification was performed with DCX^+^ immature neurons on day 7. The projection images were traced with Fiji ImageJ software; dendritic branch number and length, and soma size per cell were analyzed accordingly. Dendritic complexity was assessed by Sholl analysis, which counted the number of dendritic intersections that cross along the concentric circles spaced 10 μm from the cell body. For all of the above analyses, 50 individual DCX^+^ immature neurons were randomly chosen from five sections of the DG per mouse.

### Open field, Y maze, and contextual fear conditioning tests

The remaining animals (eight in each group) were subjected to open field, Y maze, and contextual fear conditioning tests 2 months after TBI. The Y maze test was performed 2 months after TBI surgery. The detailed protocols are provided in the Supplementary Information.

### Statistical analysis

All statistical analyses were performed using SPSS 23.0 software (SPSS Inc., Chicago, IL, USA). The Kolmogorov–Smirnov normality test was used to test the normality of data. Data with normal distribution are presented as mean ± standard deviation (SD), while data with skewed distribution are presented as medians and ranges (minimum and maximum). Comparison of means among groups was performed using one-way analysis of variance (ANOVA) with the Tukey *post-hoc* test, whereas comparison of medians among groups was performed using the Kruskal–Wallis test with Dunn's *post-hoc* test. A *P* < 0.05 was considered statistically significant.

## Results

### GDF-5 treatment promotes NSC proliferation after TBI

**Figure 1:**
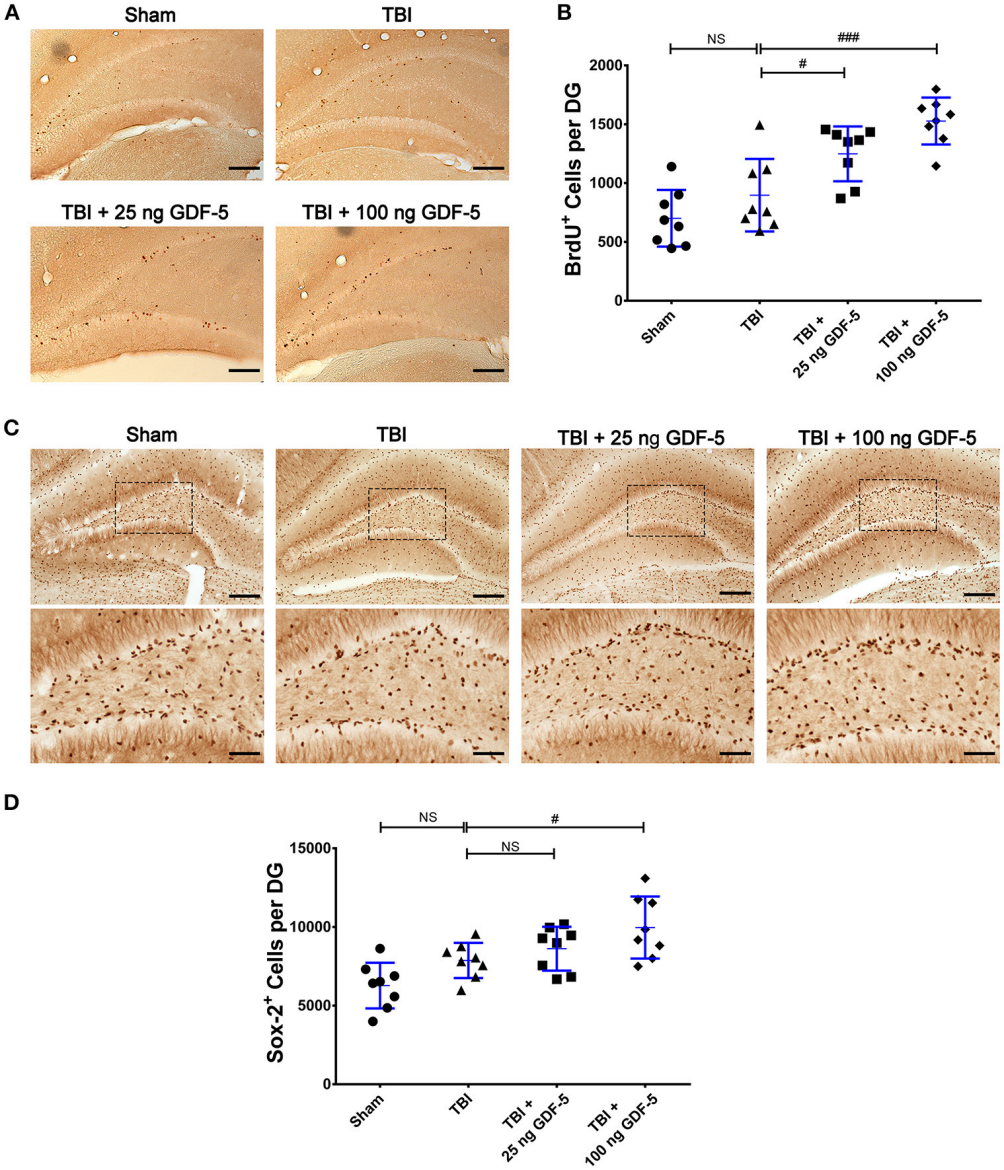
GDF-5 treatment increased the numbers of BrdU^+^ and Sox-2^+^ cells in the DG area of the hippocampus 7 days after TBI. **(A)** Representative IHC images of BrdU^+^ cells in the DG area. **(B)** Quantification of BrdU^+^ cells for all treatment groups. **(C)** Representative IHC images of Sox-2^+^ cells in the DG area (scale bars represent 100 μm). The magnified views below indicate the boxed areas of each image (scale bars represent 50 μm). **(D)** Quantification of Sox-2^+^ cells for all treatment groups. Data represent means ± SD (*n* = 8). Each dot represents the mean of quantification across five sections of the DG from an individual animal. ^#^*P* < 0.05 and ^###^*P* < 0.001, as compared with the TBI group. NS indicates no significance. ANOVA followed by Tukey's *post-hoc* test.

In the current study, we first assessed whether administration with GDF-5 promotes NSC proliferation during the subacute phase of TBI. As shown in Figure 1A, BrdU^+^ cells could be detected in all experimental groups 7 days following TBI, which were mainly located in the SGZ of the DG. Quantification showed that there was a statistically significant difference in BrdU^+^ cell counting among all groups as determined by one-way ANOVA (*P* < 0.001) (Figure 1B). As compared with the Sham group, the number of BrdU^+^ cells had increased in the TBI model group, although there was no statistically significant difference between both groups. When animals were treated with a dose of 25 or 100 ng GDF-5 after TBI, the BrdU^+^ cell count in the DG was significantly higher than that in the TBI model group. Taken together, these findings suggest that single dosing with GDF-5, 25, or 100 ng per animal, may promote NSC proliferation during the subacute phase of TBI.

**Figure 2:**
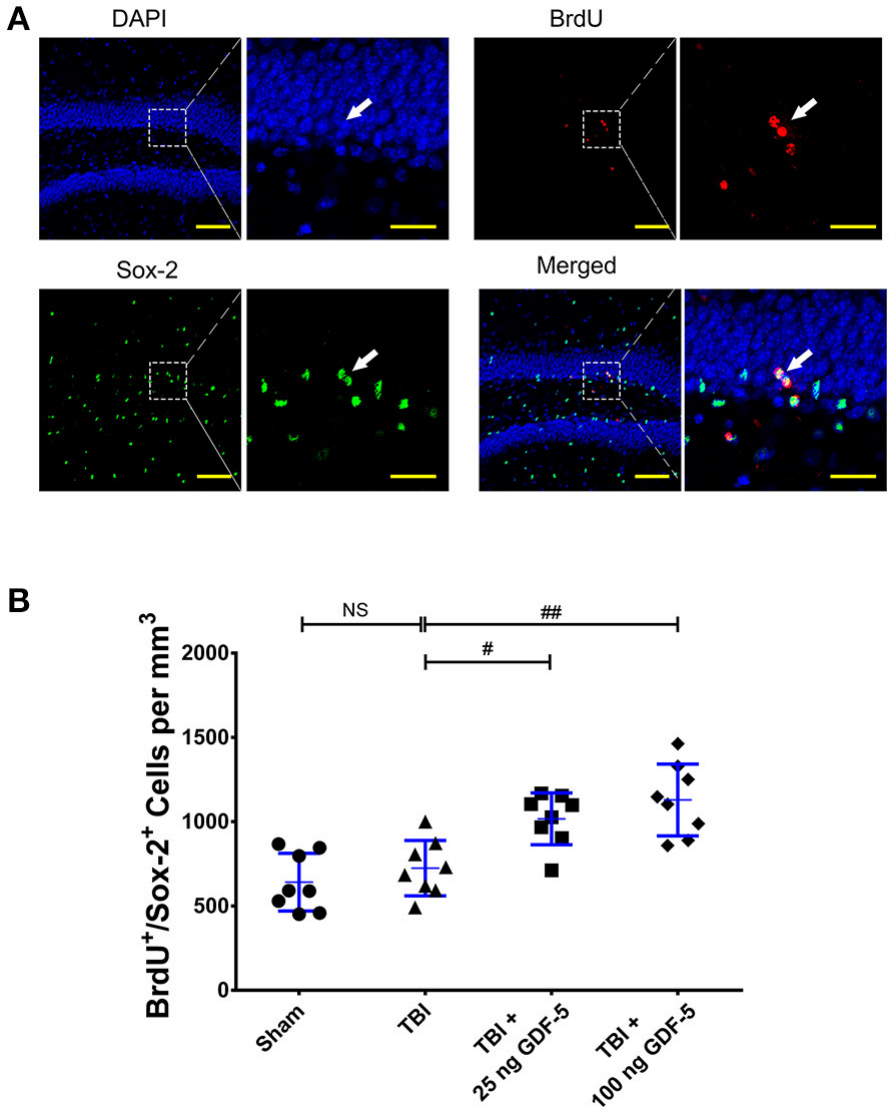
GDF-5 treatment increased the number of BrdU^+^/Sox-2^+^ cells in the DG area 7 days after TBI. **(A)** Representative confocal images (scale bars represent 100 μm). Magnified views on the right indicate the boxed areas of each image (scale bars represent 25 μm). White arrows identify BrdU and Sox-2 double-labeled cells. **(B)** Quantification of BrdU^+^/Sox-2^+^ cells for all treatment groups. Data represent means ± SD (*n* = 8). Each dot represents the mean of quantification of every sixth serial sections from an individual animal. ^#^*P* < 0.05 and ^##^*P* < 0.01, as compared with the TBI group. ANOVA followed by Tukey's *post-hoc* test.

Next, we compared the IHC results of Sox-2, an NSC marker, among all experimental groups 7 days after TBI. For both GDF-5 treatment groups, the Sox-2^+^ cell count was significantly greater in the 100 ng GDF-5 treated group than in the TBI model group (*P* < 0.05), although this was not the case for the 25 ng GDF-5 treated group (Figures 1C,D). To further validate the effects of GDF-5 on NSC proliferation, we co-localized Sox-2 in BrdU^+^ in the DG sections by IF staining. Quantitative analysis showed that the two groups administered with GDF-5 displayed a significantly higher BrdU^+^/Sox-2^+^ cell density than the TBI model group (*P* < 0.001) (Figures 2A,B). These data, combined with those shown in Figure 1, provide additional evidence that treatment with GDF-5 can potentially induce NSC proliferation during the subacute phase of TBI.

### GDF-5 treatment increases the number of immature neurons after TBI

To further investigate the neuroprotective effects of GDF-5, we conducted DCX IHC staining to examine whether post-injury treatment of GDF-5 could stimulate NSC differentiation and increase the number of immature neurons in the hippocampus.

**Figure 3:**
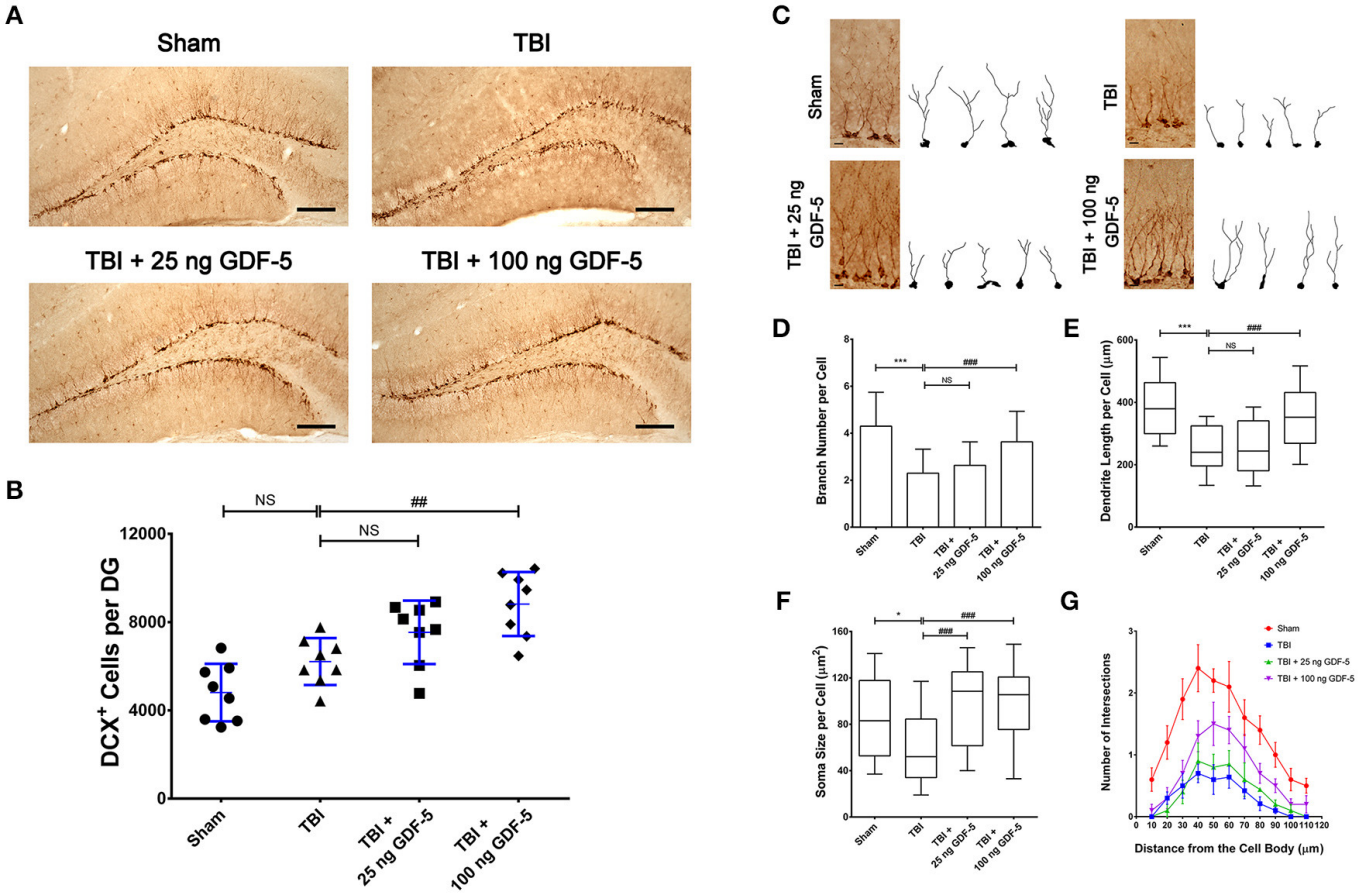
GDF-5 treatment increased the number and dendrite arborization of DCX-labeled cells in the DG area of the hippocampus 7 days after TBI. **(A)** Representative IHC images of DCX^+^ cells in the DG area. **(B)** Quantification of DCX^+^ cells for all treatment groups. Scale bars represent 100 μm. Data represent means ± SD (*n* = 8). Each dot represents the mean of quantification across five sections of the DG from an individual animal. ^##^*P* < 0.01, as compared with the TBI group. ANOVA followed by Tukey's *post-hoc* test. **(C)** Representative IHC images and projection images of DCX-labeled cells. **(D–G)** Quantification of branch number **(D)**, dendritic length **(E)**, soma size **(F)**, and dendritic complexity by Sholl analysis **(G)** of DCX-labeled cells. Scale bars represent 10 μm. For all analyses, 50 individual cells were randomly chosen from five sections of the DG per animal. Histograms and dot/line plots represent mean and SD values. Box-and-whisker plots represent median and range. ^*^P < 0.05 and ^***^P < 0.001, as compared with the Control group; ^###^*P* < 0.001, as compared with the TBI group. NS indicates no significance. ANOVA followed by Tukey's *post-hoc* test or nonparametric Kruskal–Wallis test.

As shown in Figure 3A, in all experimental groups most DCX-labeled cells were observed in the granular cell layer (GCL) of the DG, with their dendrites projecting into the molecular layer. The results of quantification revealed a significant effect of GDF-5 on density of DCX-expressing cells in the DG (*P* < 0.001). In the TBI model group, the density of DCX^+^ cells had slightly increased, as compared with the Sham group (Figure 3B), which may reflect a compensatory response to the injury. For the 25 ng GDF-5 treated groups, although the density of DCX-labeled cells was higher than that in the model group, no statistically significant difference was observed. However, the group administered with 100 ng GDF-5 displayed a significantly higher DCX^+^ cell count as compared to the TBI model group (*P* < 0.01). Collectively, these data indicated that post-injury treatment with GDF-5 has the potential to increase the immature neuron count in the DG of the hippocampus, which may be attributed to the proliferation-promoting effects of GDF-5 in NSCs.

### GDF-5 treatment increases dendrite arborization of immature neurons after TBI

To evaluate the effects of GDF-5 on dendrite development in immature neurons, we then reconstructed dendritic arbors of individual immature neurons and performed quantification of dendrite branch number, dendritic length, and soma size, as well as Sholl analysis of the dendritic complexity for all experimental groups. As presented in Figure 3C, the morphology of DCX^+^ cells in the TBI group was markedly different from that in the Sham group, evidenced by much fewer branches, shorter dendrites, and smaller somas of these cells. In contrast, in the 100 ng GDF-5 treated group, DCX-labeled cells showed similar morphology to those from the Sham group, although this effect was not significant in the group treated with 25 ng GDF-5. These results indicate that a high dose of GDF-5 may exert a protective effect on immature neurons against TBI-induced dendrite degeneration, or stimulate dendrite regrowth following TBI.

Quantification results are summarized in Figures 3D–G. After TBI, the average number of dendrite branches, and average dendritic length and soma size were significantly decreased in the model group as compared to the Sham group (Figures 3D–F), which is consistent with the findings of TBI-induced dendritic impairment presented in Figure 3C. In the group treated with 100 ng GDF-5, this impairment was dramatically improved, manifested by more dendritic braches per cell, as well as greater dendritic length and soma size. Sholl analysis (Figure 3G) revealed that compared with the sham controls, immature neurons in the TBI model group showed reduced dendritic complexity in response to the injury; while post-injury treatment with high dose of GDF-5 effectively counteracted this reduction, particularly evident at a distance of 40–80 μm. Taken together, these data indicated a potential neuroprotective effect of GDF-5 against dendrite degeneration following TBI.

### GDF-5 treatment prevents decreased p-CREB after TBI

**Figure 4:**
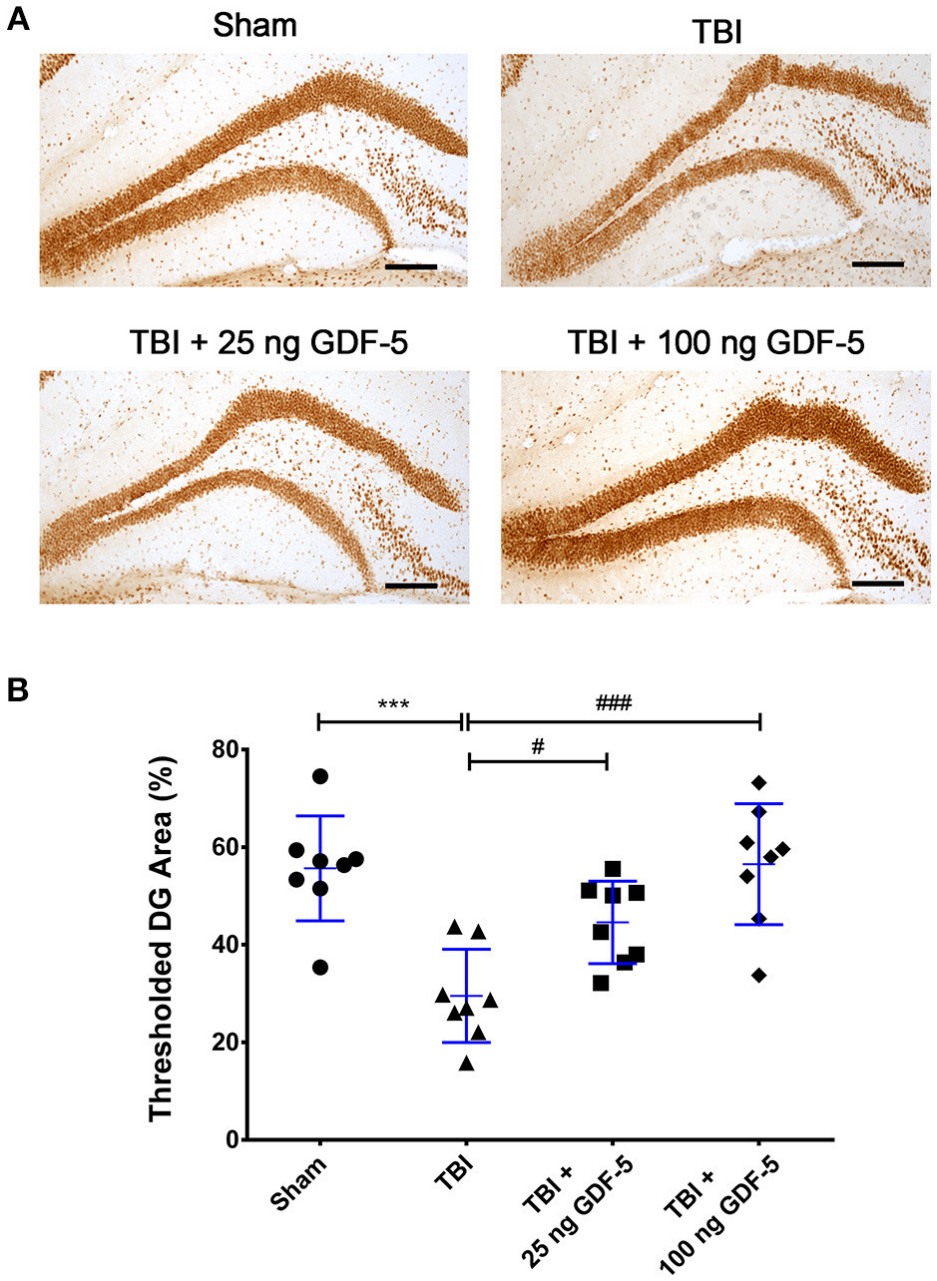
GDF-5 treatment prevents the decrease in p-CREB after TBI. **(A)** Representative IHC images of p-CREB staining in the DG area of the hippocampus. **(B)** Quantification of p-CREB immunoreactivity. Scale bars represent 100 μm. Data represent means ± SD (*n* = 8). Each dot represents the mean of quantification across five sections of the DG from an individual animal. ^***^P < 0.001, as compared with the Control group; ^#^*P* < 0.05 and ^###^*P* < 0.001, as compared with the TBI group. ANOVA followed by Tukey's *post-hoc* test.

To identify the potential molecular mechanisms responsible for the neuroprotective effects of GDF-5, we examined the activation of the CREB signaling pathway, which has been documented to play a critical role in NSC differentiation, neuronal survival, and dendritic growth (19). Seven days after TBI, we conducted IHC staining for p-CREB and compared the immunoreactivity in all experimental groups. As shown in Figures 4A,B, directly following TBI the signal of p-CREB in the DG was significantly decreased as compared to sham brains. In the two GDF-5 treated groups, however, this decrease was prevented, and a dose-dependent effect could be observed. Particularly, animals administered with 100 ng GDF-5 showed a similar p-CREB level in the DG as compared to sham controls. These results indicate that GDF-5 prevents TBI-induced dephosphorylation of CREB in the DG of injured mouse brain.

### GDF-5 treatment enhances activation of newborn neurons

**Figure 5:**
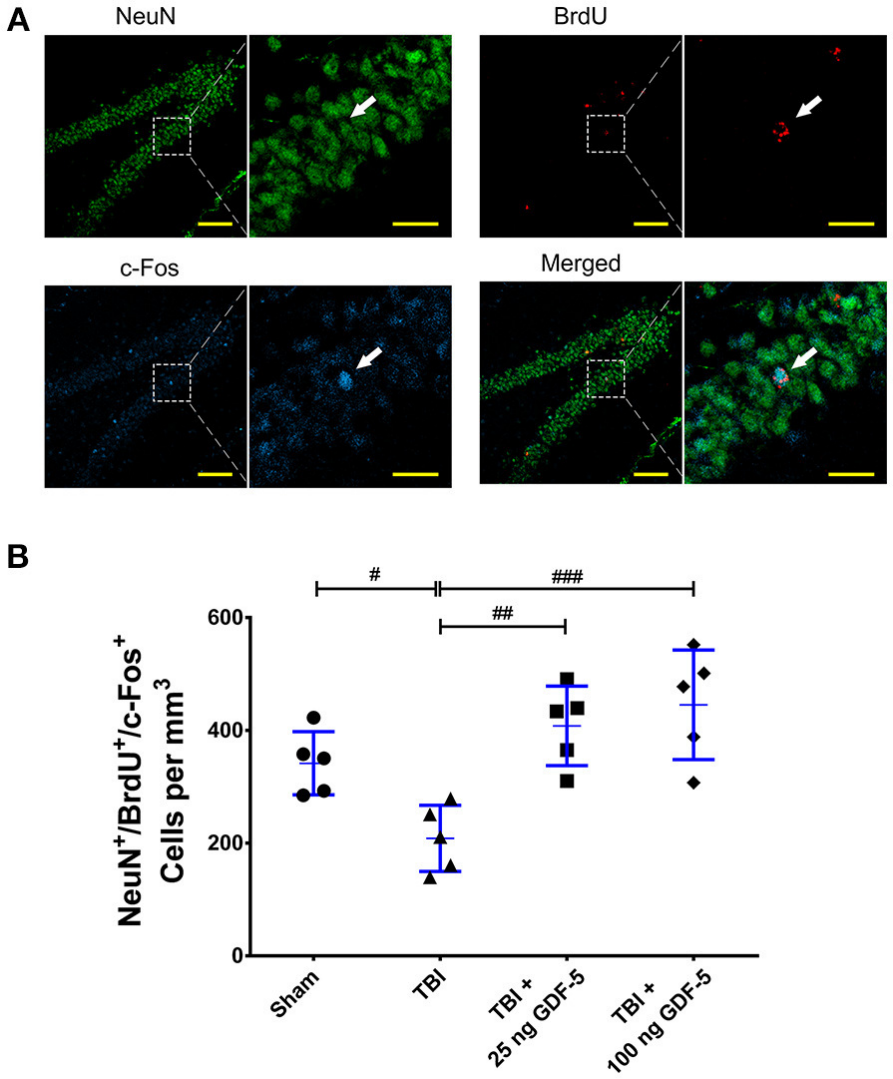
GDF-5 treatment enhanced activation of newborn neurons in the DG after TBI. **(A)** Representative confocal images of NeuN^+^/BrdU^+^/c-Fos^+^ cells in the hippocampal DG area 35 days after TBI (scale bars represent 100 μm). Magnified views on the right indicate the boxed areas of each image (scale bars represent 25 μm). White arrows identify NeuN, BrdU, and c-Fos triple-labeled cells. **(B)** Quantification of NeuN^+^/BrdU^+^/c-Fos^+^ cells for all treatment groups. Data represent means ± SD (*n* = 5). Each dot represents the mean of quantification of every sixth serial sections of the DG from an individual animal. ^#^*P* < 0.05, ^##^*P* < 0.01, and ^###^*P* < 0.001, as compared with the TBI group. ANOVA followed by Tukey's *post-hoc* test.

To investigate whether GDF-5 treatment enhances activation of newborn neurons, we co-localized c-Fos, a marker of newly excited neurons (20, 21), in BrdU^+^/NeuN^+^ cells in the DG sections using IF staining. As presented in Figures 5A,B, the quantitative evaluation revealed a considerable difference among all groups in the density of NeuN^+^/BrdU^+^/c-Fos^+^ cells (*P* < 0.001). The TBI model group displayed a significantly lower NeuN^+^/BrdU^+^/c-Fos^+^ cell count than the Sham group. In the two GDF-5 treated groups, however, the number of NeuN^+^/BrdU^+^/c-Fos^+^ cells had remarkably increased, indicating that more newborn neurons are active overall in the DG of GDF-5-treated animals.

### GDF-5 treatment improves cognitive impairments after TBI

**Figure 6:**
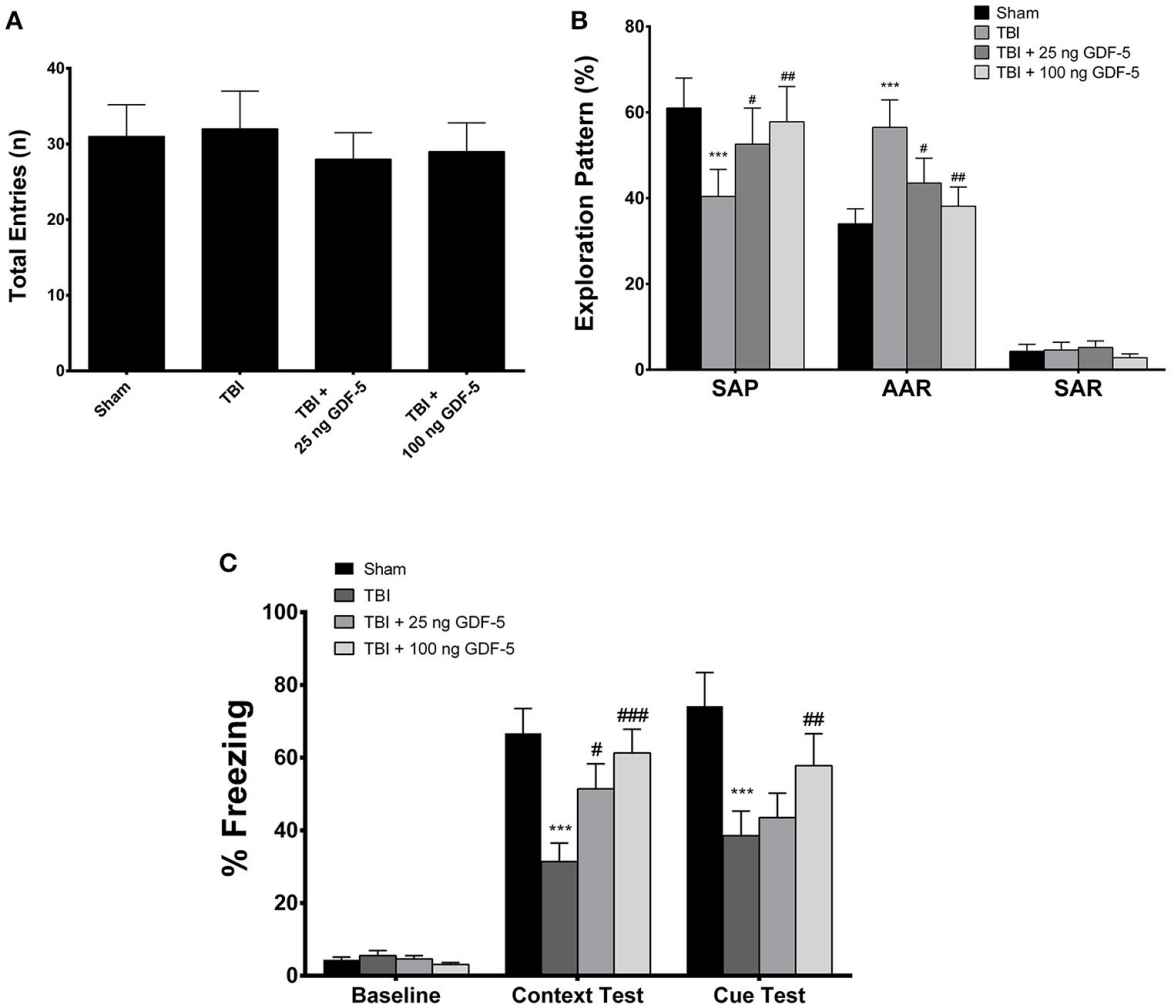
GDF-5 treatment improved spatial reference memory and hippocampus-dependent working memory of animals, as assessed by Y maze and contextual fear conditioning tests, respectively. **(A)** There was no significant difference in total number of entries in the Y maze among all groups. **(B)** Animals receiving 25 or 100 ng GDF-5 made significantly more SAPs and fewer AARs. **(C)** Freezing behavior was recorded and analyzed. Treatment with 25 or 100 ng GDF-5 could improve contextual freezing response. All data are presented as means ± SD (*n* = 8). ^***^P < 0.001, as compared with the Control group; ^#^*P* < 0.05, ^##^*P* < 0.01, and ^###^*P* < 0.001, as compared with the TBI group. ANOVA followed by Tukey's *post-hoc* test. SAP, spontaneous alternation performance; AAR, alternate arm return; SAR, same arm return.

To examine whether treatment with GDF-5 improves cognitive and behavioral impairments following TBI, we measured spatial memory in all experimental groups with a Y-maze and hippocampus-dependent working memory with a contextual and cued fear conditioning procedure at 2 months after injury. The results are presented in Figures 6A–C.

Prior to the Y-maze study, the locomotor activity of all animals was measured using the open field test. As shown in Supplementary Figures 2A–C, all groups displayed similar locomotor activity throughout the experiment. In the Y-maze test, there was no significant difference in total entries across all groups (Figure 6A). Nevertheless, mice in the TBI model group made fewer spontaneous alternation performances (SAPs) and more alternate arm returns (AARs) than sham animals, indicating a cognitive impairment caused by TBI (Figure 6B). In the two GDF-5-treated groups, however, animals made significantly more SAPs and fewer AARs. Particularly, the numbers of SAPs and AARs in the high dose group were comparable to those in the Sham group (Figure 6B). In the contextual and cued fear conditioning test (Supplementary Figure 3), animals displayed a considerably weaker contextual freezing response following TBI than did the sham control mice, suggesting a deficit in spatial working memory. However, this deficit could be improved by post-injury administration of GDF-5, particularly evident in the group receiving 100 ng GDF-5 (Figure 6C). Collectively, the results of the Y-maze and fear conditioning tests demonstrate the recovery of hippocampus-dependent cognitive and behavioral functions in GDF-5 treated animals.

## Discussion

It is well-documented that GDF-5 is expressed in the developing central nervous system (CNS) and transmits its signals via binding to bone morphogenetic protein (BMP) receptors type IB (BMPRIB) and II (BMPRII), which subsequently regulates diverse biological processes within various neural cell types (22, 23). In this study, our data showed that after administration of GDF-5, the numbers of BrdU^+^, Sox-2^+^, BrdU^+^/Sox-2^+^, and DCX^+^ cells were considerably increased in TBI-injured animals, which could indicate the potential neurostimulating effects of GDF-5. These findings were closely in line with observations in previous studies (24, 25). However, it should be noted that under TBI conditions, immature neurons are particularly vulnerable, which consequently leads to a compensatory increase in the generation of immature neurons (26). In the present study, we did find a slight increase in the numbers of BrdU^+^, Sox-2^+^, BrdU^+^/Sox-2^+^, and DCX^+^ cell groups 7 days following TBI, but this change was not statistically significant. Hence, regarding the significantly increased numbers of these immunolabeled cells in the DG of GDF-5-treated animals, the supportive signals produced by administered GDF-5 may be the leading contributor. In the microenvironment of the DG, administered GDF-5 activates its specific receptors to induce direct or indirect neurotrophic effects, consequently promoting proliferation of NSCs and their differentiation into immature neurons. Nevertheless, it should be addressed here that Sox-2- and DCX-expressing cell populations are highly heterogeneous (e.g., Sox-2-expressing cells can be divided into Type I NSCs and Type IIa and IIb NPCs, while DCX-expressing cells consist of proliferating neuroblasts and post-mitotic immature neurons) (27), which warrants a further study to better understand the effects of GDF-5 on various subpopulations of these cells. Furthermore, in post-TBI neurogenesis, immature neurons may be born either before or after injury. Although our results indicate that GDF-5 treatment can increase the number of immature neurons after TBI, we still cannot rule out the possibility that GDF-5 enhances the survival of immature neurons born prior to the injury.

Dendritic arborization is a key neuronal differentiation process and a direct prerequisite for complex neuronal communication (28). In TBI microenvironment, however, dendrite development in immature neurons is affected (29, 30). GDF-5 has been demonstrated to be a key physiological regulator of dendrite growth during development (31). In this study, Sholl analysis showed that treatment with GDF-5 increases dendrite arborization of immature neurons after TBI. This effect is most likely due to activation of the Smad signaling pathway by binding of GDF-5 to its receptors, consequently turning on downstream signals that promote dendrite growth (31). At the molecular level, phosphorylated Smads 1/5/8 allow them to form a complex with Smad 4, subsequently interacting with effectors of other signaling pathways [e.g., protein kinase A (PKA)] to mediate several physiological responses elicited by TGF-β, including the activation of CREB signaling, and then regulate neural functions and behavior (32, 33).

The transcription factor CREB is well-known to regulate neural plasticity and memory processes in the brain (34). It is activated upon phosphorylation either by PKA and/or Ca^2+^/calmodulin-dependent protein kinases, thereby targeting genes and molecules important for adaptive neuronal responses as well as for complex neural functions, such as learning and long-term memory formation (33). Under TBI conditions, chronic signaling deficit in CREB activation occurs in the hippocampus, manifested by a decrease in basal levels of phosphorylated CREB over several months post-trauma (35). In our study, we found that following TBI, the phosphorylation level of CREB in the DG was significantly decreased, while this change was counteracted by treatment with GDF-5, which may, at least in part, mechanistically be related to the enhanced activation of PKA resulting from improved Smad signaling in response to exogenous GDF-5 administration. Nevertheless, it should be noted that after the primary TBI there are a number of secondary injury factors that contribute to neuronal cell loss and dysfunction, such as oxidative stress and excitotoxicity (36, 37). Thus far, we still do not know how these factors lead to reduced CREB signaling and how GDF-5 could counteract the detrimental effects related to these factors. Further investigation is therefore needed to address these issues.

Cognitive and behavioral deficits are common challenges occurring in TBI patients (38). Accumulating evidence suggests that neuronal cell loss and compromised neuronal integrity in the hippocampus underlie cognitive deficits following TBI (39). In our study, however, the TBI model group did not show any difference or even a slight increase in the density of DCX^+^ immature neurons in the DG as compared to the Sham group. This may be due to injury-induced neurogenesis, which is presumably a compensatory response to the injury. Nevertheless, 35 days after modeling TBI, we found that the TBI model group displayed a significantly lower NeuN^+^/BrdU^+^/c-Fos^+^ cell count than the Sham group, suggesting that fewer immature neurons survived to become functional mature neurons in the post-TBI microenvironment, which may consequently contribute to memory impairment. In our Y-maze and fear conditioning tests, the injured mice did exhibit impairments in hippocampus-dependent learning and memory paradigms, but these deficits could be improved by treatment with GDF-5. These observations further suggest the beneficial effects of GDF-5, which may be derived from the hippocampal neurogenesis promoted by GDF-5 during the subacute phase of TBI. Nevertheless, one limitation to the current study was that we did not assess the maturation of immature neurons following GDF-5 treatment, which would be critical to better understand the improvement in behavioral tests. Additionally, as the underlying cellular mechanisms of cognition and memory formation in the hippocampus are extremely complicated and remain poorly elucidated, we do not yet understand the detailed cellular processes involved in reconsolidating such complex neural functions by GDF-5-induced hippocampal neurogenesis and thus our findings merit further investigation.

In summary, our data reveals that direct injection of GDF-5 into the hippocampus can stimulate neurogenesis and improve functional recovery in a mouse TBI model, indicating that increasing the hippocampal level of GDF-5 may be a promising approach against neuronal loss and cognitive and behavioral deficits following TBI. Although the low dose of GDF-5 showed limited efficacy and direct administration of GDF-5 into the brain is invasive and may cause an immune response, our findings still provide substantial insights into the potential merits of GDF-5 and its downstream effects for clinical application in the treatment of TBI.

## Author contributions

HW and TC conceived and designed the study. HW, JL, DX, and QZ carried out experiments. HW, DX, and QZ analyzed the data. HW and TC wrote the manuscript. All authors read and approved the final manuscript.

### Conflict of interest statement

The authors declare that the research was conducted in the absence of any commercial or financial relationships that could be construed as a potential conflict of interest. The reviewer JP and handling Editor declared their shared affiliation.

## Abbreviations

BrdU: 5-bromo-2′-deoxyuridine
AAR: Alternate arm return
BMP: Bone morphogenetic protein
BMPRIB: BMP receptor type IB
BMPRII: BMP receptor type II
CREB: cAMP response element binding protein
CS: Conditioned stimulus
CCI: Controlled cortical impact
DG: Dentate gyrus
DCX: Doublecortin
GCL: Granular cell layer
GDF-5: Growth differentiation factor 5
IHC: Immunohistochemical
KA: Kainic acid
NPC: Neural progenitor cell
NSC: Neural stem cell
ANOVA: One-way analysis of variance
PKA: Protein kinase A
SAR: Same arm return
SAP: Spontaneous alternation performance
SD: Standard deviation
SEM: Standard error of the mean
SGZ: Subgranular zone
TGF-β: Transforming growth factor beta
TBI: Traumatic brain injury
US: Unconditioned stimulus.
