# Supplementary material for: Growth Differentiation Factor 5 Improves Neurogenesis and Functional Recovery in Adult Mouse Hippocampus Following Traumatic Brain Injury
Source: Front Neurol. 2018 Jul 23;9:592. doi: 10.3389/fneur.2018.00592 (PMC6064945; doi:10.3389/fneur.2018.00592)
Supplement: Supplementary file 1 [file Data_Sheet_1.DOCX]

# Supplementary Information

# Growth differentiation factor 5 improves neurogenesis and functional recovery in adult mouse hippocampus following traumatic brain injury

# *Running Title: GDF-5 for TBI Treatment*

Hongjie Wu; Jing Li; Dongxiao Xu; Qiansheng Zhang; Tao Cui*

Department of Neurology, the First Affiliated Hospital and College of Clinical Medicine of Henan University of Science and Technology, Luoyang, Henan Province, China, 471003

*Corresponding author: Tao Cui, M.D., Associate Professor of Medicine

Affiliation of corresponding author: Department of Neurology, the First Affiliated Hospital and College of Clinical Medicine of Henan University of Science and Technology, Luoyang, Henan Province, China, 471003

Address of corresponding author: Guanlin Road and Xuefang Street (at the intersection), Luolong District, Luoyang, Henan Province, China, 471003

Tel: +86-379-69823256

Fax: N/A

Email: lycuitao@outlook.com

Methods

Establishment of mouse TBI model

All animals were anesthetized using isoflurane solution (2.5% for induction, 1.5% during surgery) and secured in prone position in a stereotaxic frame (Braintree Scientific, Inc., Braintree, MA, USA). The craniotomy was perform in the right hemisphere. In brief, under sterile conditions, the skin was incised and then a 3.5 - 4 mm craniotomy was performed lateral to the midline and midway between the bregma and lambda. The skullcap was carefully removed to expose cortical tissue without disruption of the dura. For animals in all model groups, controlled cortical impact TBI was generated using an electromagnetic force-controlled impactor (Shanghai Yuyan Instruments, Inc., Shanghai, China). Before induction of TBI, the tip of the impactor (3 mm in diameter) was angled and kept perpendicular to the exposed cortical surface. The contact velocity and deformation depth was set at 3.0 m/sec and 1.0 mm, respectively. For animals in the Sham group, no further manipulation was performed after the incision was made.

Open field test

The remaining animals (eight in each group) were subjected to open field, Y maze, and contextual fear conditioning tests two months after TBI. All behavioral tests were conducted during light cycle. For the open field test, a chamber with dimensions of 40 cm length ×40 cm width × 35cm height (AniLab Inc., Ningbo, China) was used as the testing apparatus. The light intensity of the chamber was 200 lux. Animals were placed in the center of the chamber and recorded for 5 min. Total distance traveled (cm), time spent in the center area (20 × 20 cm), and vertical activity (defined as using the upper and lower limbs to reach up the side of the chamber and hold body up) were measured using the AniLab Software for Locomotor Activity (AniLab Inc.). Between animals, the chamber was cleaned of debris and olfactory cues with 70% ethanol (v/v). All testing work was performed blindly.

Y maze test

The apparatus for Y maze test consisted of two symmetrical plastic arms (dimension: 15.5 cm length ×7.5 cm width × 12.5 cm height) and one longer plastic arm at 120° angles (dimension: 20.7 cm length ×7.7 cm width ×12.7 cm height). Upon onset of the experiment, mice were placed at the end of the longer arm and allowed to freely explore the three arms for 5 min. An arm entry was recorded when all four limbs of a mouse were inside an arm. The maze was cleaned with 70% ethanol (v/v) before the experiment and between testing animals to eliminate traces of odor. The number of total arm entries and the number of different triads (including spontaneous alternation performance (SAP), alternate arm return (AAR), and same arm return (SAR), which are defined as a set of consecutive arm entries (1)) were recorded. The percentage of each exploration pattern was calculated by dividing the total number of triads by the number of each exploration pattern. All testing work was performed blindly.

Contextual fear conditioning test

The procedures for contextual fear conditioning test were followed in accordance with the previous literature (2). Conditioned fear was displayed as freezing behavior. Prior to the experiment, a program-controlled chamber was set up so that light and tone cue (conditioned stimulus, CS) and mild foot shock (unconditioned stimulus, US) could be available during the test. On the first day (training day), animals were placed in the chamber for a 3-min baseline recording followed by four CS-US pairings with 60 sec each pair. The parameters for CS and US were: CS duration 20 sec, sound level 70 dB, 2 kHz; US duration 2 sec, US intensity 0.7 mA. US was delivered at the end of CS. On the second day, each animal was placed in the same chamber containing the same context as the first day, but did not receive any CS or US. Freezing was analyzed for 4 min. One hour later, animals were placed in a new context with a different odor (3% acetic acid, v/v), floor texture, and chamber walls and shape. Animals were allowed to explore for 3 min before they were re-exposed to CS for another 3 min. Freezing was defined as the absence of all movement except that related to respiration (2). Freezing was measured using a video tracking system and software (ANY-maze, Wood Dale, IL, USA).

References

1. Holter SM, Garrett L, Einicke J, et al. Assessing Cognition in Mice. *Curr Protoc Mouse Biol.* 2015;**5**:331-358.
2. Curzon P, Rustay NR, Browman KE. Cued and Contextual Fear Conditioning for Rodents. In: Buccafusco JJ, ed. *Methods of Behavior Analysis in Neuroscience.* 2nd ed. Boca Raton (FL)2009.

Supplementary Figure 1


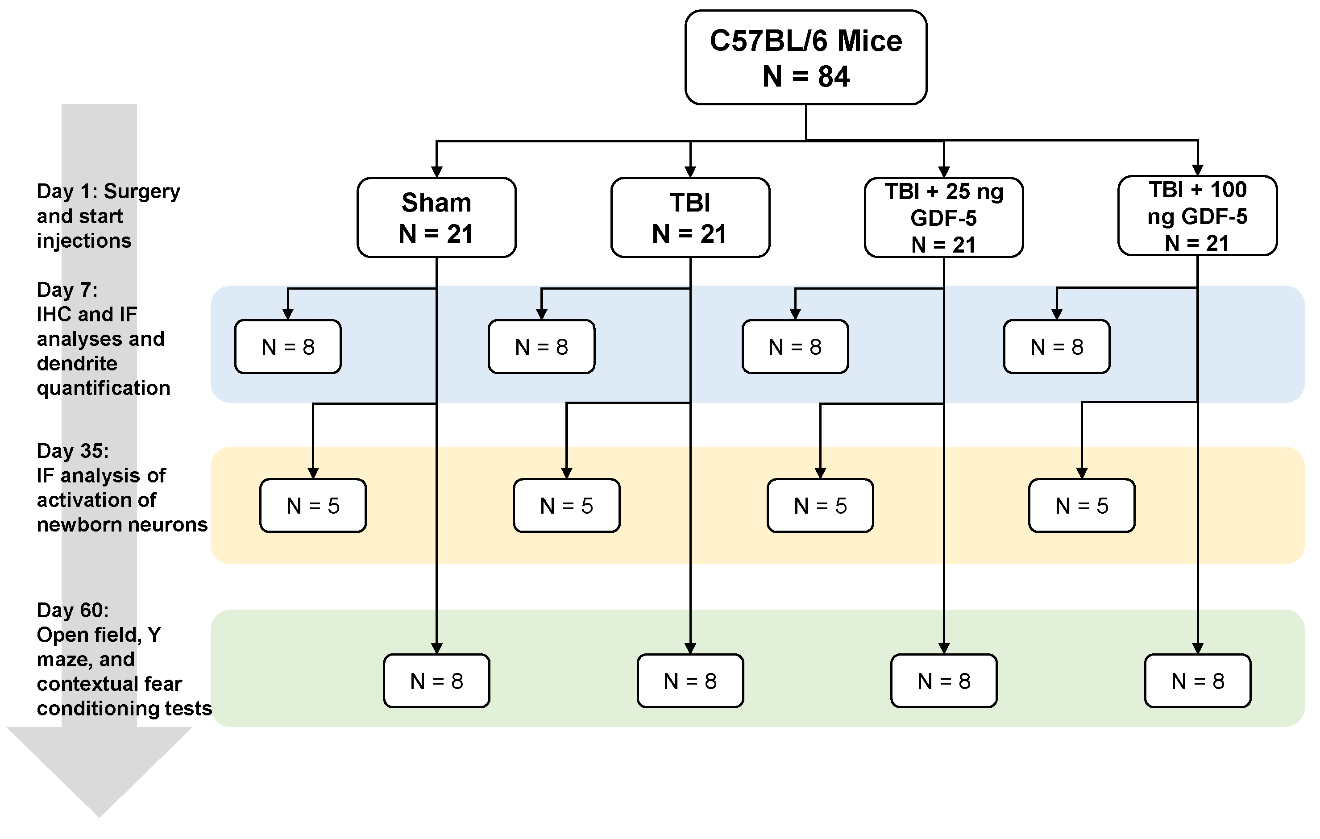


**Figure 1**. Diagram of the treatment groups and number of animals within each group. TBI, traumatic brain injury; GDF-5, growth differentiation factor 5; IHC, immunohistochemical; IF, immunofluorescence

Supplementary Figure 2

**
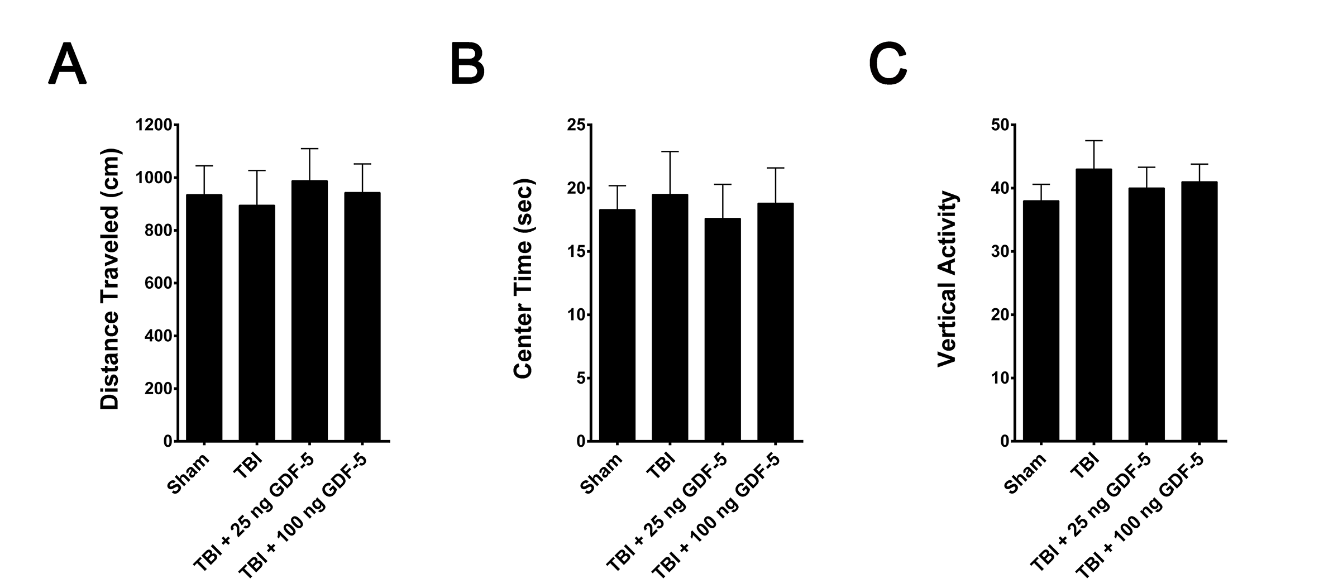
**

**Figure 2**. The open field test revealed that there were no significant differences in total distance traveled (A), center time (B), and vertical activity (C), indicating that all groups had similar locomotor activity.. Data represent means ± SD (n = 8). ANOVA followed by Tukey’s post hoc test.

Supplementary Figure 3

**
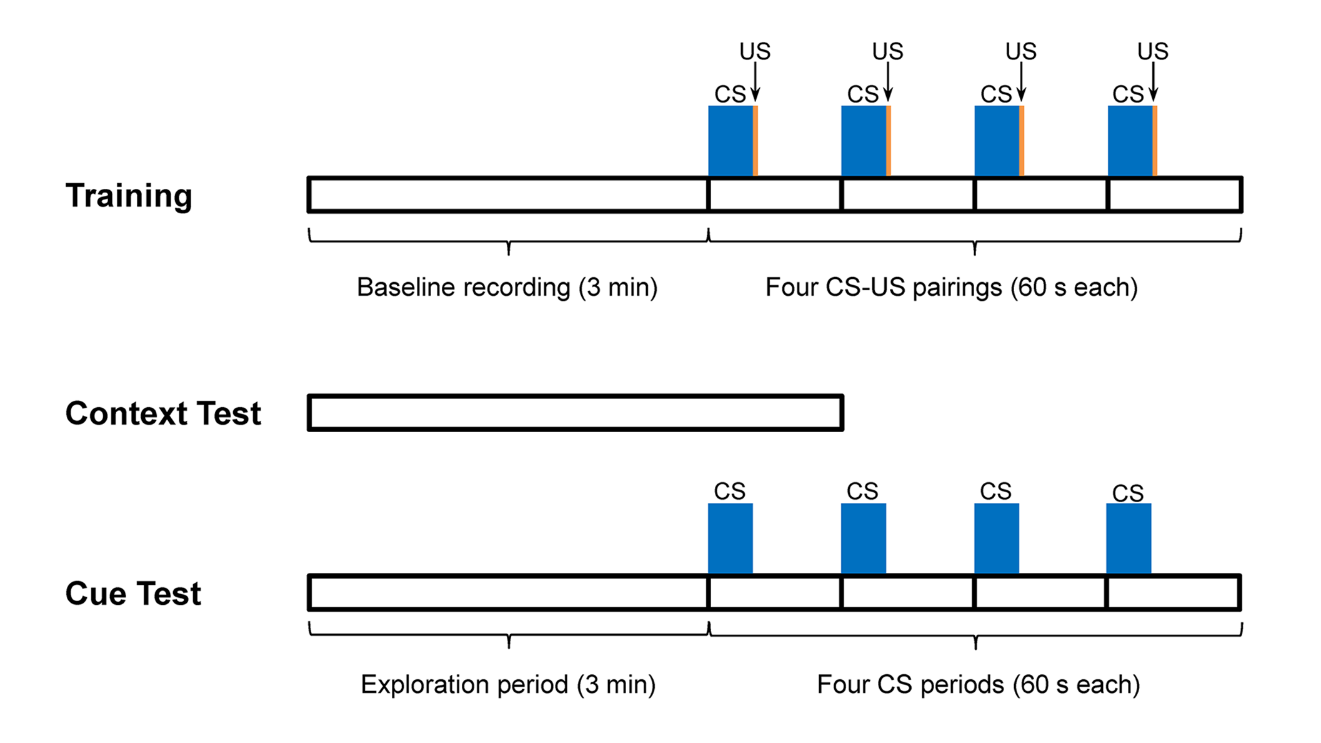
**

**Figure 3**. The test paradigm for fear conditioning.
